# Correction: Student-centered factors influencing inclusion in biomedical majors among first-year undergraduate students

**DOI:** 10.1371/journal.pone.0322259

**Published:** 2025-04-04

**Authors:** The PLOS One Staff

## Notice of republication

This article was republished on March 20, 2025, to correct an error in the author byline that was introduced during the typesetting process. The publisher apologizes for this error. Please download the article again to view the corrected version. The originally published, uncorrected article and the republished, corrected article are provided here for reference.

## Supporting information

S1 File Originally published, uncorrected article. (PDF)

S2 File Republished, corrected article. (PDF)
