# Supplementary material for: Student-centered factors influencing inclusion in biomedical majors among first-year undergraduate students
Source: PLoS One. 2024 Dec 31;19(12):e0312862. doi: 10.1371/journal.pone.0312862 (PMC11687708; doi:10.1371/journal.pone.0312862)
Supplement: S1 Table — (DOCX) [file pone.0312862.s001.docx]

S1 Table. Multinomial Regression Model Results

|  | **Univariate Results** | | | | | **Multivariate Results** | | | |
| --- | --- | --- | --- | --- | --- | --- | --- | --- | --- |
| **Factor** | **Outcome** | **N** | **OR^1^** | **95% CI^1^** | **p-value** | **Outcome** | **OR^1^** | **95% CI^1^** | **p-value** |
| Science Identity** | Persistence | 7,252 | 0.55 | 0.50, 0.59 | <0.001 | Persistence | 0.57 | 0.51, 0.63 | <0.001 |
|  | Pushout | 7,252 | 0.25 | 0.23, 0.27 | <0.001 | Pushout | 0.51 | 0.46, 0.57 | <0.001 |
|  | To | 7,252 | 0.61 | 0.54, 0.68 | <0.001 | To | 0.67 | 0.57, 0.79 | <0.001 |
| Science Self-Efficacy^#,^** | Persistence | 7,252 | 0.84 | 0.78, 0.90 | <0.001 | Persistence | 1.17 | 1.07, 1.28 | <0.001 |
|  | Pushout | 7,252 | 0.51 | 0.48, 0.54 | <0.001 | Pushout | 0.96 | 0.88, 1.04 | 0.3 |
|  | To | 7,252 | 0.77 | 0.69, 0.86 | <0.001 | To | 0.90 | 0.79, 1.03 | 0.13 |
| Science Career ^#,^** | Persistence | 7,252 | 0.66 | 0.61, 0.71 | <0.001 | Persistence | 0.86 | 0.79, 0.94 | 0.001 |
|  | Pushout | 7,252 | 0.25 | 0.23, 0.27 | <0.001 | Pushout | 0.40 | 0.37, 0.44 | <0.001 |
|  | To | 7,252 | 0.75 | 0.67, 0.84 | <0.001 | To | 0.99 | 0.86, 1.14 | >0.9 |
| Less than 20 years | Persistence | 7,252 | 1.02 | 0.68, 1.52 | >0.9 | Persistence | 1.18 | 0.78, 1.80 | 0.4 |
|  | Pushout | 7,252 | 0.70 | 0.52, 0.94 | 0.018 | Pushout | 0.76 | 0.53, 1.10 | 0.14 |
|  | To | 7,252 | 1.94 | 0.85, 4.45 | 0.12 | To | 1.99 | 0.86, 4.61 | 0.11 |
| Female ^#,^** | Persistence | 7,252 | 0.98 | 0.85, 1.13 | 0.8 | Persistence | 0.92 | 0.79, 1.07 | 0.3 |
|  | Pushout | 7,252 | 1.27 | 1.13, 1.43 | <0.001 | Pushout | 1.13 | 0.97, 1.31 | 0.12 |
|  | To | 7,252 | 1.08 | 0.86, 1.35 | 0.5 | To | 1.12 | 0.89, 1.42 | 0.3 |
| LGBTQ ** | Persistence | 7,252 | 0.99 | 0.80, 1.23 | >0.9 | Persistence | 0.96 | 0.77, 1.20 | 0.7 |
|  | Pushout | 7,252 | 1.71 | 1.46, 1.99 | <0.001 | Pushout | 1.41 | 1.16, 1.72 | <0.001 |
|  | To | 7,252 | 1.14 | 0.84, 1.56 | 0.4 | To | 1.06 | 0.77, 1.47 | 0.7 |
| Native English Speaker ^#,^** | Persistence | 7,252 | 0.81 | 0.68, 0.97 | 0.019 | Persistence | 0.73 | 0.60, 0.88 | 0.001 |
|  | Pushout | 7,252 | 1.26 | 1.08, 1.48 | 0.004 | Pushout | 0.93 | 0.76, 1.14 | 0.5 |
|  | To | 7,252 | 1.31 | 0.96, 1.78 | 0.092 | To | 0.84 | 0.60, 1.18 | 0.3 |
| No Financial Concerns ** | Persistence | 7,252 | 1.22 | 1.03, 1.43 | 0.020 | Persistence | 1.17 | 0.98, 1.40 | 0.074 |
|  | Pushout | 7,252 | 0.99 | 0.86, 1.14 | >0.9 | Pushout | 1.06 | 0.89, 1.27 | 0.5 |
|  | To | 7,252 | 0.52 | 0.37, 0.72 | <0.001 | To | 0.62 | 0.44, 0.88 | 0.007 |
| Pell** | Persistence | 7,252 | 1.05 | 0.92, 1.21 | 0.4 | Persistence | 0.93 | 0.81, 1.08 | 0.4 |
|  | Pushout | 7,252 | 0.93 | 0.83, 1.04 | 0.2 | Pushout | 0.93 | 0.81, 1.07 | 0.3 |
|  | To | 7,252 | 0.44 | 0.35, 0.56 | <0.001 | To | 0.53 | 0.41, 0.68 | <0.001 |
| ASIAN** | Persistence | 7,252 | 0.83 | 0.71, 0.97 | 0.022 | Persistence | 0.90 | 0.73, 1.11 | 0.3 |
|  | Pushout | 7,252 | 0.58 | 0.50, 0.66 | <0.001 | Pushout | 0.46 | 0.38, 0.57 | <0.001 |
|  | To | 7,252 | 0.43 | 0.32, 0.59 | <0.001 | To | 0.26 | 0.18, 0.36 | <0.001 |
| BLACK** | Persistence | 7,252 | 1.13 | 0.95, 1.35 | 0.2 | Persistence | 1.11 | 0.85, 1.45 | 0.5 |
|  | Pushout | 7,252 | 0.67 | 0.57, 0.78 | <0.001 | Pushout | 0.63 | 0.48, 0.83 | 0.001 |
|  | To | 7,252 | 0.49 | 0.35, 0.69 | <0.001 | To | 0.28 | 0.17, 0.44 | <0.001 |
| HISPANIC ^#,^** | Persistence | 7,252 | 1.17 | 0.99, 1.38 | 0.061 | Persistence | 1.01 | 0.80, 1.29 | >0.9 |
|  | Pushout | 7,252 | 1.15 | 1.01, 1.32 | 0.035 | Pushout | 0.65 | 0.52, 0.81 | <0.001 |
|  | To | 7,252 | 0.73 | 0.55, 0.97 | 0.032 | To | 0.40 | 0.28, 0.58 | <0.001 |
| Multiracial/AIAN ^#,^** | Persistence | 7,252 | 1.19 | 0.98, 1.43 | 0.079 | Persistence | 1.23 | 0.97, 1.56 | 0.081 |
|  | Pushout | 7,252 | 1.30 | 1.12, 1.52 | <0.001 | Pushout | 0.88 | 0.71, 1.09 | 0.2 |
|  | To | 7,252 | 0.90 | 0.65, 1.23 | 0.5 | To | 0.48 | 0.34, 0.68 | <0.001 |
| HBCU ^#,^** | Persistence | 7,252 | 1.34 | 1.11, 1.62 | 0.002 | Persistence | 1.55 | 1.20, 2.02 | <0.001 |
|  | Pushout | 7,252 | 0.80 | 0.67, 0.95 | 0.012 | Pushout | 1.47 | 1.11, 1.95 | 0.008 |
|  | To | 7,252 | 0.79 | 0.56, 1.11 | 0.2 | To | 1.73 | 1.11, 2.69 | 0.016 |
| MSI** | Persistence | 7,252 | 1.40 | 1.23, 1.60 | <0.001 | Persistence | 1.28 | 1.09, 1.50 | 0.002 |
|  | Pushout | 7,252 | 1.98 | 1.77, 2.21 | <0.001 | Pushout | 1.90 | 1.63, 2.22 | <0.001 |
|  | To | 7,252 | 1.01 | 0.83, 1.25 | 0.9 | To | 1.28 | 1.01, 1.62 | 0.043 |
| Interaction with Academic Advisors ^#,^** | Persistence | 7,252 | 1.01 | 0.77, 1.33 | >0.9 | Persistence | 1.25 | 0.93, 1.66 | 0.14 |
|  | Pushout | 7,252 | 0.65 | 0.53, 0.79 | <0.001 | Pushout | 1.05 | 0.81, 1.36 | 0.7 |
|  | To | 7,252 | 1.24 | 0.79, 1.97 | 0.4 | To | 1.48 | 0.92, 2.40 | 0.11 |
| Interaction with Graduate Students ^#,^** | Persistence | 7,252 | 0.74 | 0.65, 0.85 | <0.001 | Persistence | 0.87 | 0.75, 1.00 | 0.054 |
|  | Pushout | 7,252 | 0.58 | 0.52, 0.64 | <0.001 | Pushout | 0.83 | 0.72, 0.95 | 0.007 |
|  | To | 7,252 | 0.94 | 0.76, 1.16 | 0.6 | To | 0.97 | 0.77, 1.21 | 0.8 |
| Felt Isolated ^#,^** | Persistence | 7,252 | 1.11 | 1.04, 1.19 | 0.002 | Persistence | 1.06 | 0.98, 1.15 | 0.12 |
|  | Pushout | 7,252 | 1.15 | 1.09, 1.22 | <0.001 | Pushout | 1.12 | 1.04, 1.22 | 0.003 |
|  | To | 7,252 | 1.02 | 0.92, 1.14 | 0.6 | To | 0.98 | 0.87, 1.11 | 0.8 |
| Part of Campus Community ^#,^** | Persistence | 7,252 | 0.89 | 0.84, 0.95 | <0.001 | Persistence | 1.01 | 0.92, 1.10 | 0.9 |
|  | Pushout | 7,252 | 0.83 | 0.79, 0.88 | <0.001 | Pushout | 0.96 | 0.88, 1.05 | 0.4 |
|  | To | 7,252 | 0.86 | 0.78, 0.95 | 0.004 | To | 0.88 | 0.77, 1.01 | 0.076 |
| Faculty Concern about Progress ^#,^** | Persistence | 7,252 | 1.05 | 0.98, 1.12 | 0.2 | Persistence | 1.15 | 1.06, 1.24 | 0.001 |
|  | Pushout | 7,252 | 1.01 | 0.96, 1.07 | 0.7 | Pushout | 1.03 | 0.95, 1.11 | 0.5 |
|  | To | 7,252 | 0.89 | 0.81, 0.99 | 0.025 | To | 0.95 | 0.84, 1.07 | 0.4 |
| Valued at Institution** | Persistence | 7,252 | 0.86 | 0.81, 0.92 | <0.001 | Persistence | 0.89 | 0.81, 0.97 | 0.007 |
|  | Pushout | 7,252 | 0.96 | 0.91, 1.02 | 0.2 | Pushout | 1.08 | 0.99, 1.18 | 0.088 |
|  | To | 7,252 | 0.99 | 0.89, 1.09 | 0.8 | To | 1.12 | 0.97, 1.28 | 0.11 |
| Faculty Show Interest ^#,^** | Persistence | 7,252 | 0.92 | 0.86, 0.99 | 0.016 | Persistence | 0.95 | 0.87, 1.03 | 0.2 |
|  | Pushout | 7,252 | 1.03 | 0.98, 1.09 | 0.3 | Pushout | 1.17 | 1.08, 1.27 | <0.001 |
|  | To | 7,252 | 0.88 | 0.79, 0.97 | 0.011 | To | 0.89 | 0.79, 1.01 | 0.069 |
| Sense of Community with Students ^#,^** | Persistence | 7,252 | 0.98 | 0.92, 1.05 | 0.5 | Persistence | 1.12 | 1.02, 1.22 | 0.012 |
|  | Pushout | 7,252 | 0.89 | 0.84, 0.94 | <0.001 | Pushout | 1.03 | 0.94, 1.12 | 0.5 |
|  | To | 7,252 | 0.97 | 0.88, 1.08 | 0.6 | To | 1.10 | 0.96, 1.26 | 0.2 |
| Job Responsibilities Conflict* | Persistence | 7,252 | 1.08 | 1.01, 1.16 | 0.017 | Persistence | 1.09 | 1.02, 1.17 | 0.012 |
|  | Pushout | 7,252 | 1.00 | 0.95, 1.06 | 0.9 | Pushout | 1.08 | 1.01, 1.16 | 0.034 |
|  | To | 7,252 | 1.02 | 0.92, 1.13 | 0.8 | To | 1.05 | 0.94, 1.17 | 0.4 |
| Found Balance with School and Responsibilities ^#,^** | Persistence | 7,252 | 0.91 | 0.85, 0.97 | 0.004 | Persistence | 0.96 | 0.89, 1.03 | 0.2 |
|  | Pushout | 7,252 | 1.00 | 0.94, 1.05 | 0.9 | Pushout | 1.04 | 0.96, 1.12 | 0.3 |
|  | To | 7,252 | 1.03 | 0.93, 1.14 | 0.6 | To | 1.05 | 0.93, 1.18 | 0.4 |
| Graduate Degree Planned** | Persistence | 7,252 | 0.91 | 0.75, 1.10 | 0.3 | Persistence | 0.95 | 0.78, 1.16 | 0.6 |
|  | Pushout | 7,252 | 0.47 | 0.41, 0.54 | <0.001 | Pushout | 0.56 | 0.47, 0.66 | <0.001 |
|  | To | 7,252 | 0.65 | 0.50, 0.86 | 0.002 | To | 0.71 | 0.53, 0.94 | 0.017 |
| Professional Degree Planned** | Persistence | 7,252 | 0.48 | 0.39, 0.58 | <0.001 | Persistence | 0.59 | 0.48, 0.73 | <0.001 |
|  | Pushout | 7,252 | 0.10 | 0.08, 0.11 | <0.001 | Pushout | 0.21 | 0.17, 0.25 | <0.001 |
|  | To | 7,252 | 0.42 | 0.31, 0.55 | <0.001 | To | 0.57 | 0.42, 0.78 | <0.001 |
| Other Degree Planned ^#,^** | Persistence | 7,252 | 1.03 | 0.53, 1.98 | >0.9 | Persistence | 1.12 | 0.57, 2.21 | 0.7 |
|  | Pushout | 7,252 | 0.38 | 0.21, 0.68 | 0.001 | Pushout | 0.71 | 0.36, 1.42 | 0.3 |
|  | To | 7,252 | 1.02 | 0.41, 2.52 | >0.9 | To | 1.45 | 0.57, 3.70 | 0.4 |
| ^1^OR = Odds Ratio, CI = Confidence Interval | | | | | | | | | |
