# Supplementary material for: Student-centered factors influencing inclusion in biomedical majors among first-year undergraduate students
Source: PLoS One. 2024 Dec 31;19(12):e0312862. doi: 10.1371/journal.pone.0312862 (PMC11687708; doi:10.1371/journal.pone.0312862)
Supplement: S2 Table — (DOCX) [file pone.0312862.s002.docx]

S2 Table. Logistic Regression Model Results

|  | **Univariate Results** | | | **Multivariate Results** | | |
| --- | --- | --- | --- | --- | --- | --- |
| **Characteristic** | **OR^1^** | **95% CI^1^** | **p-value** | **OR^1^** | **95% CI^1^** | **p-value** |
| Science Identity^#,**^ | 0.47 | 0.43, 0.51 | <0.001 | 0.92 | 0.81, 1.04 | 0.2 |
| Science Self-Efficacy^**^ | 0.62 | 0.58, 0.67 | <0.001 | 0.82 | 0.74, 0.90 | <0.001 |
| Science Career^**^ | 0.41 | 0.38, 0.45 | <0.001 | 0.50 | 0.45, 0.55 | <0.001 |
| Less than 20 years | 0.69 | 0.45, 1.03 | 0.078 | 0.62 | 0.38, 0.98 | 0.043 |
| Female* | 1.29 | 1.10, 1.51 | 0.001 | 1.25 | 1.04, 1.51 | 0.016 |
| LGBTQ^**^ | 1.72 | 1.39, 2.14 | <0.001 | 1.42 | 1.11, 1.82 | 0.005 |
| Native English Speaker* | 1.56 | 1.28, 1.89 | <0.001 | 1.29 | 1.02, 1.64 | 0.036 |
| No Financial Concerns ^#,*^ | 0.82 | 0.68, 0.98 | 0.026 | 0.96 | 0.78, 1.19 | 0.7 |
| Pell | 0.88 | 0.76, 1.02 | 0.092 | 1.06 | 0.89, 1.26 | 0.5 |
| ASIAN** | 0.70 | 0.58, 0.84 | <0.001 | 0.50 | 0.39, 0.65 | <0.001 |
| BLACK** | 0.59 | 0.48, 0.72 | <0.001 | 0.57 | 0.41, 0.80 | 0.001 |
| HISPANIC ^#,**^ | 0.99 | 0.83, 1.18 | 0.9 | 0.62 | 0.47, 0.81 | <0.001 |
| Multiracial/AIAN ^#,**^ | 1.10 | 0.90, 1.35 | 0.3 | 0.69 | 0.53, 0.90 | 0.006 |
| HBCU ^#,**^ | 0.59 | 0.48, 0.74 | <0.001 | 0.91 | 0.66, 1.27 | 0.6 |
| MSI** | 1.41 | 1.22, 1.63 | <0.001 | 1.57 | 1.30, 1.89 | <0.001 |
| Interaction with Advisors ^#,**^ | 0.64 | 0.48, 0.84 | 0.002 | 0.87 | 0.63, 1.20 | 0.4 |
| Interactions with Grad ^#,**^ | 0.78 | 0.67, 0.90 | <0.001 | 0.96 | 0.81, 1.14 | 0.7 |
| Felt Isolated | 1.04 | 0.97, 1.11 | 0.3 | 1.05 | 0.95, 1.15 | 0.3 |
| Part of Campus | 0.93 | 0.87, 1.00 | 0.059 | 0.96 | 0.86, 1.06 | 0.4 |
| Faculty Concerned | 0.97 | 0.90, 1.04 | 0.3 | 0.91 | 0.83, 1.00 | 0.057 |
| Valued at Institution** | 1.12 | 1.04, 1.20 | 0.003 | 1.21 | 1.09, 1.35 | <0.001 |
| Faculty Show Interest** | 1.12 | 1.04, 1.20 | 0.002 | 1.20 | 1.09, 1.32 | <0.001 |
| Sense of Community** | 0.91 | 0.85, 0.98 | 0.009 | 0.90 | 0.81, 0.99 | 0.035 |
| Job Conflicts ^#,*^ | 0.92 | 0.86, 0.99 | 0.031 | 0.97 | 0.89, 1.06 | 0.5 |
| Balance with Job* | 1.09 | 1.02, 1.17 | 0.013 | 1.11 | 1.01, 1.21 | 0.026 |
| Graduate Degree Planned** | 0.52 | 0.43, 0.62 | <0.001 | 0.60 | 0.49, 0.73 | <0.001 |
| Professional Degree Planned** | 0.20 | 0.16, 0.25 | <0.001 | 0.35 | 0.27, 0.44 | <0.001 |
| Other Degree Planned ^#,**^ | 0.37 | 0.18, 0.76 | 0.006 | 0.74 | 0.33, 1.68 | 0.5 |
| ^1^OR = Odds Ratio, CI = Confidence Interval | | | | | | |
