# Supplementary material for: Correction: Student-centered factors influencing inclusion in biomedical majors among first-year undergraduate students
Source: PLoS One. 2025 Apr 4;20(4):e0322259. doi: 10.1371/journal.pone.0322259 (PMC11970658; doi:10.1371/journal.pone.0322259)
Supplement: S1 File — (PDF) [file pone.0322259.s001.pdf]

RESEARCH ARTICLE

Student-centered factors influencing inclusion in biomedical majors among first-year undergraduate students

Perceived fit and social integration writing group, NIH-BUILD¶*

¶ Complete membership of the author group can be found in the Acknowledgments

* kala.mehta@ucsf.edu

Abstract

The ability to maintain a diverse scientific workforce is vital to promoting the US's economic and technological competitiveness. Data have shown disparities in science, mathematics, medical, and engineering programs across each level of education from high school to doctoral studies for students from underrepresented groups (URG). Research suggests that many URG students are pushed out of the biomedical track early in their academic careers, particularly during the first year. Most of these studies focus on well-known indicators, such as science identity and research self-efficacy, to study inclusion in biomedical majors. The current study sought to understand the influence of institutional environment and student-based characteristics on changes in major during the first-year undergraduate experience. Overall, these results indicate that institutional factors have an impact alongside student-based factors in biomedical major retention in the first year. This manuscript identifies actions that institutions can take to improve biomedical major retention.

OPEN ACCESS

Citation: Perceived fit and social integration writing group, NIH-BUILD (2024) Student-centered factors influencing inclusion in biomedical majors among first-year undergraduate students. PLoS ONE 19(12): e0312862. https://doi.org/10.1371/journal.pone.0312862

Editor: Claudia Noemi González Brambila, Instituto Tecnologico Autonomo de Mexico, MEXICO

Received: January 19, 2024

Accepted: October 14, 2024

Published: December 31, 2024

Copyright: © 2024 Perceived fit and social integration writing group, NIH-BUILD. This is an open access article distributed under the terms of the Creative Commons Attribution License, which permits unrestricted use, distribution, and reproduction in any medium, provided the original author and source are credited.

Data Availability Statement: De-identified data is publicly available in repository at URL https://github.com/awagler2/firstyearSTEMM.git.

Funding: The authors prepared this manuscript on behalf of the Diversity Program Consortium. Work reported in this publication was supported by the Office of The Director, National Institutes of Health Common Fund and Office of Scientific Workforce Diversity awards UL1GM118979, UL1GM118976, UL1GM118973, UL1GM118964, UL1GM118985,

Introduction

Maintaining a diverse scientific workforce is vital to promoting the nation's economic competitiveness and position in technological leadership [1–3]. Valuing diversity of perspectives, experiences, and knowledge creation allows us to leverage opportunities for creativity and fully exchange ideas. Hong and Page [4] demonstrated that problem-solving within diverse groups is more effective than in non-diverse groups, suggesting a role for diversity in improving the quality of our scientific research output [5]. The United States of America (US) has yet to achieve the goal of diversifying the workforce within science, technology, engineering, mathematics, and medicine (biomedical) research [6]. Despite attempts to increase interest in pursuing biomedical careers, undergraduate degree completion in biomedical majors remains low [7–9]. In biomedical fields, the growing shortage of biomedical professionals (such as physicians and other healthcare workers) was exacerbated by the COVID-19 pandemic [10, 11]. In the US, approximately half of undergraduates who intend to major in biomedical fields leave within their first two years, with few non biomedical majors switching to biomedical majors [8]. This situation is worse for students from underrepresented groups (URG).

UL1GM118991, UL1GM118982, UL1GM118988, UL1GM118970, UL1GM118967, and U54GM119024 administered by the National Institute of General Medical Sciences. The work is solely the responsibility of the authors and does not necessarily represent the official view of the National Institutes of Health. Individual NIH BUILD projects are funded by linked grants through the NIH Common Fund which includes the following projects- SF BUILD: UL1 GM118985; TL4 GM118986; RL5GM118984; STEM BUILD PODER: RL5GM118975 TL4GM118977, UL1GM118976; UMBC BUILD: 5RL5GM118987; 5TL4GM118989; 5UL1GM118988 (LEO); ReBUILDetroit RL5GM118981, TL4GM118983, UL1GM118982; CSULB BUILD: NIGMS UL1GM118979, TL4GM118980, RL5GM118978; UTEP BUILDing SCHOLARS: RL5GM118969, TL4GM118971, and UL1GM118970; BUILD EXITO: RL5GM118963, TL4GM118965, and UL1GM118964.

Competing interests: The authors have declared that no competing interests exist.

Referred to as the "leaky pipeline" or the "pipeline problem" to a biomedical career, [12, 13] data have shown disparities in science and engineering education across each level from high school to doctoral studies, directly leading to a less diverse biomedical workforce. Remarkably, the achievement gap, measured by the percentage of degrees awarded, widens as students move along their academic stages. In the US, students across most racial and ethnic groups enter STEM majors, including biomedical, at similar rates: [14, 15] approximately 30% of American Indian/Alaskan Native, 54% of Asian, 40% of Black or African American, 45% of Latinx, and 40% of White undergraduate students enter college intending to major in a biomedical field [15]. Yet, there remains a disparity within the US regarding biomedical degree attainment, with Black or African American, American Indian/Alaskan Native, and Latinx students disproportionately being pushed out of biomedical majors compared to White and Asian students [15, 16].

Pushout refers to practices that contribute to students dropping out [17]. According to the National Institutes of Health (NIH), URG in biomedical majors include individuals who identify with racial and ethnic backgrounds that are historically underrepresented in the biomedical sciences (American Indian/Alaskan Native, Black or African American, Latinx, those from under-resourced backgrounds, and individuals with disabilities), e.g., [3, 18–20]. The Diversity Program Consortium (DPC) was established to enhance diversity in the biomedical research workforce it compiled a list of undergraduate majors and fields of study that would further that goal and determined the scope of the Enhance Diversity Study. The current study uses the biomedical majors defined in the Enhance Diversity Study.

Research suggests that many students from URG are pushed out of biomedical fields early in their academic careers [17, 21–23]. In a study of six institutions, Weston [22] found that the majority of students who switch majors do so early, with half switching by the end of the first year and another 30% switching by the end of the second year. Further, Riegle-Crumb et al. [16] found that Black or African American and Latinx students are significantly more likely than their White peers to switch and earn a degree in another field. Understanding factors that contribute to student persistence and reduce biomedical pushout is critical. Using the term pushout rather than dropping out acknowledges extrinsic factors contributing to this outcome [17].

Despite the biomedical pushout that occurs, many students persist and pursue graduate training in biomedical-related fields, such as biomedical research and non-research careers [24, 25]. In a longitudinal study of within-field career changes in the biomedical sciences, Rosenzweig and colleagues [25] found that most students (84%) stayed in a biomedical major, and 16% switched to a non-biomedical major. More importantly, they found that of those who remained in a biomedical major, nearly half (46%) had changed their career plans, with women more likely to change their career plans to ones that needed fewer years of education [25].

Current study focus

Biomedical degree programs and classrooms are perceived as deliberately created exclusionary spaces where students must prove they deserve to stay [26]. Although most students face challenges navigating these spaces, URG students experience them while potentially being burdened with unfounded stereotypes about presumed inferior cognitive and mathematical ability [16, 27, 28]. Research suggests that feelings of prejudice, alienation, and rejection negatively correlate with URG students' persistence [29]. The measures that we include regarding inclusion and culture in biomedical majors are regarded as "key hallmarks" by NIH, [30] namely science identity (the personal feeling that students are scientists and that others see them as scientists [31]), research self-efficacy (a students' self-appraisal about abilities to

complete tasks related to biomedical discipline demands [32]), and sense of belonging (students' perceptions of social support, connections, and acceptance by others on campus [33]).

There is ample evidence of the importance of science identity [34–40] and research self-efficacy [33, 41–59] for students from URG entering, persisting, and achieving academic success in biomedical fields. Not only are these factors important in and of themselves, but they are also impacted by day-to-day relational factors experienced by students. Thus, we also explore issues that focus more on the actions of faculty, advisors, and institutions, such as student interactions with faculty and student perceptions of the campus community [60–62]. We label these as "modifiable" factors since they are external influences on the first-year student experience and can be modified by leadership and faculty in an institutional setting. Consequently, our study seeks to identify modifiable institutional environment- and student-based characteristics as potential intervention targets that may influence science identity, research self-efficacy, and academic self-concept during students' first year of undergraduate study.

Where possible, we include additional student-based non-modifiable factors (i.e., socioeconomic status, race and ethnicity, sex, first-generation student status, disability status) known to influence these self-perceptions. The current study reviews outcomes of persistence in, or pushout of biomedical-related majors at the end of the first year of college. During their first year of college, student interactions with faculty have been linked to academic performance, faculty contact satisfaction, and overall college satisfaction [63]. This potentially modifiable set of factors is more salient for URG first-year students due to their relative lack of the requisite educational and research experiences to develop a conceptually clear sense of science identity and research self-efficacy [19]. Hence, we focus on these factors for the measurement of inclusion, which will be emphasized in the analysis.

In sum, few studies examine factors contributing to the persistence or pushout of biomedical majors in their first year and focus on factors that can prevent the pushout of URG populations in biomedical majors. Hence, our study focuses on the first-year experiences to understand the role of institutional environment and student characteristics early in the undergraduate experience. The following overarching question guided the study, "How are sociodemographic characteristics, relational factors, and science identity, research self-efficacy, and sense of belonging associated with biomedical major choice for first-year college students: 1) persisted in biomedical major, 2) pushed out of biomedical major, 3) became a biomedical major, and 4) never a biomedical major?".

Materials and methods

The data presented here come from the Enhance Diversity Study, a longitudinal evaluation of training programs funded as part of the NIH Diversity Program Consortium.

Participants

Every Fall term from 2015 through 2019, first-year students from 11 universities (California State University, Long Beach; California State University, Northridge; Morgan State University; Portland State University; San Francisco State University; University of Alaska, Fairbanks; University of Detroit Mercy; University of Maryland, Baltimore County; University of Texas at El Paso; Wayne State University; and Xavier University of Louisiana) involved with the NIH's Building Infrastructure Leading to Diversity (BUILD) initiative were invited to participate in the Enhance Diversity Study. Consent was informed in written form. No parents or guardians were contacted in the consent process since all participants were 18 or older. The recruitment period was the Summer and Fall of each year, beginning in Summer 2015. Follow-up surveys were administered each Spring, concluding in the Spring of 2019 for the

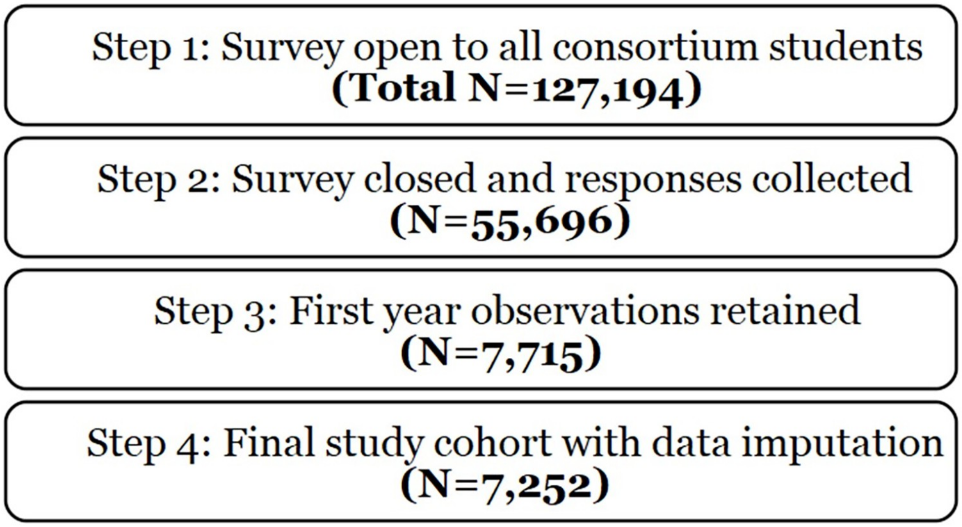

Fig 1. Consort diagram of the data collection process.

https://doi.org/10.1371/journal.pone.0312862.g001

students enrolled in the 11 BUILD- affiliated universities. A total of 32,963 students completed a survey at the beginning and end of their first year [64]. The analysis included students who completed a survey in the Fall at the beginning of their first year and in the Spring at the end of their first year. Out of this group of students, we restricted the analysis to include full-time and first-year students, who accounted for 94% of the data available, resulting in a total of 7,252 research participants. See Fig 1 for a description of the data collection process.

Ethical approvals and study registration

The Office of the Human Research Protection Program (OHRPP) at UCLA reviewed and approved all the surveys used for the Enhance Diversity Study. HERI's The Freshman Survey (TFS) was approved on 6th November 2014 by the UCLA Institutional Review Board (UCLA IRB), Protocol 10-001293.The HERI Your First College Year Survey (YFCY) was approved on 7th December 2015 by UCLA IRB Protocol 15–001776. The Enhance Diversity Study Student Annual Follow Up Survey (SAFS) was approved on 18th October 2016 by UCLA IRB, Protocol 16–000046. A waiver of signed consent was approved for the entire study. A written information sheet was available for paper surveys. For online surveys, students actively indicated consent to participate on an introduction screen before proceeding to the survey.

Institutional setting

The relevant institutional and program characteristics of institutions involved in this study are provided in Table 1. Note that all institutions are affiliated with the NIH BUILD program, and most are ~~a~~ majority-minority serving institutions, meaning that more than 50% of the students enrolled in those campuses are not White.

Procedure

Students were recruited either through an invitation to participate as an incoming freshman/first-year student or by participation in one or more of the BUILD activities offered at their institution. The following validated surveys were administered to collect the data presented in this paper. The Fall survey, HERI's *The Freshmen Survey* (TFS), was administered by each

Table 1. University level characteristics of 10 NIH BUILD sites, 2016.

Program	Public/ Private	Undergraduate Enrollment	Minority- Serving Type	Majority-Minority	Freshmen Entry into BUILD Program	Percent of Pell ** Recipients
CSULB	Public	32,200	HSI, AANAPISI	Yes	No	54%
CSUN	Public	35,500	HSI, AANAPISI	Yes	No	57%
MSU	Public	6,300	HBCU	Yes	No	55%
PSU	Public	22,500	AANAPISI*	No	No	39%
SF State	Public	25,900	HSI, AANAPISI	Yes	No	55%
UAF	Public	7,200	AANAPISI	No	Yes	24%
UDM	Private	2,600	None	No	Yes	32%
UMBC	Public	11,100	None	No	Yes	30%
UTEP	Public	23,900	HSI	Yes	Yes	52%
Xavier	Private	4,500	HBCU	Yes	No	17%

* pathway partners were AANAPISI; AANAPISI: Asian American and Native American Pacific-Islander Serving Institution, HBCU: Historically Black College or University, HSI: Hispanic-Serving Institution, Majority-Minority: The majority of the undergraduate students come from historically classified minority groups; Source: U.S. Department of Education, *Distribution of Federal Pell Grant Program Funds by Institution*, https://www2.ed.gov/finaid/prof/resources/data/pell-institution.html

https://doi.org/10.1371/journal.pone.0312862.t001

institution in-person, online, or both, and the administration mode could vary from year to year. The Spring surveys, *Your First College Year* (YFCY) or *Student Annual Follow-Up Survey* (SAFS), were administered online.

The TFS (baseline survey) was administered through the Higher Education Research Institute (HERI) [65]. Two different surveys were administered at the end of the first year (in the Spring). In Spring 2016, the study used the YFCY survey (administered through HERI). Beginning in Spring 2017, the EDS designed the SAFS, a survey specifically focused on constructs important to the DPC evaluation. HERI permitted using items from the YFCY on the SAFS for continuity in data collection. Specific measures from these surveys used in this analysis are outlined below. When the recoding of variables was indicated, it was described along with the descriptions of the measures.

Measures

Sociodemographic factors. Various baseline (Fall) survey items were used to characterize respondents. Age was calculated by subtracting the survey completion date from the date of birth reported by students. This approximate age is included in model analyses, while the percentage of students younger than 20 is reported for descriptives.

Items related to finances were also assessed. Students were asked if they had none, some, or major concerns about their ability to finance their college education; this item was entered into the model as a numerical variable. Socioeconomic status was measured using self-reported participants' Pell Grant status.

Native language was identified by asking, "Is English your native language?" Students responded yes or no (no was the reference group for analysis). Race and /ethnicity were derived from an item asking, "Are you:" followed by a list of 13 options (with mark all that apply format). Separate dummy variables were entered into the models for American Indian/Alaskan Native, Asian, Black/African American, Latinx, and Multiracial/Other (White was the reference group). In analyses, American Indian/Alaskan Native was combined with Multiracial/Other due to small sample sizes.

Sex was ascertained by a single item, "Your sex with response options of: Male or Female. The Male group was the reference group used in analyses. In later versions of the survey, other

categories for gender were included, but for consistency across years, we used the variable for sex in this study. Sexual orientation was ascertained with the question, "What is your sexual orientation?". In 2015–2017, response options were Heterosexual/straight, Gay, Lesbian, Bisexual, Queer and Other. In 2018 and 2019, the response options Pansexual and Asexual were added to the item, and "Other" was changed to "Not listed above." The final variable used in analyses is a binary variable, with non-LGBTQ as the reference group.

Biomedical inclusion factors. The biomedical major inclusion factors examined in this study are science identity and research self-efficacy. These psychosocial constructs associated with first-year college experiences were defined using item-response theory. Each construct was scored and then transformed on a scale of 0–100.

Science identity was assessed using four items to ascertain how much students see themselves as scientists. They rated to what extent each statement was true for them, using a 5-point Likert scale response format, ranging from Strongly disagree (1) to Strongly agree (5). A sample item includes, "I think of myself as a scientist."

For research self-efficacy, students rated their confidence in carrying out scientific research. The stem question, "How confident are you that you can:" was followed by 10 items, with five response options, ranging from 1 = Not at all to 5 = Absolutely. For example, "Explain the results of a study."

A third inclusion factor was science career interest, assessed with one item, "Will you pursue a science-related research career?" A 5-point response option ranged from Definitely no to Definitely yes.

Institutional environment factors. Institution type was coded for each school included in the dataset. Race and ethnicity breakdown of the student population at each institution was identified to ascertain if the school was an Asian American and Native American Pacific Islander serving institution (AANAPISI), Hispanic serving institution (HSI), Historically Black College or University (HBCU), minority-majority institution (where more than 50% of students were from racial and ethnic minoritized groups), or none of the above. Since each institution could have multiple designations, dummy variables for each type were created and included in the model. However, the indicator variable for AANAPISI was dropped from the models due to the sparsity of data in this group. The following variables were included as institutional environment factors: faculty interactions, campus satisfaction, and college experience.

Interactions with different persons on campus were measured with a stem item: "Since entering this college, how often have you interacted with the following people:". Response options were, 1 = Never; 2 = 1 or 2 times per term; 3 = 1 or 2 times per month; 4 = Once a week; 5 = 2 or 3 times per week; 6 = Daily. The current analysis included items for academic advisors and graduate students.

A measure of campus satisfaction was included that indicates an overall sense of community among students with response options, 1 = Can't Rate/No Experience; 2 = Very Dissatisfied; 3 = Dissatisfied; 4 = Neutral; 5 = Satisfied; 6 = Very Satisfied. The experiences of first-year college students were measured by asking how often students felt a particular experience, with the following response options, 1 = Not At All; 2 = Occasionally; 3 = Frequently. One item included in the current analysis was "Isolated from campus life." Other opinions about campus experiences were assessed by asking students to rate how strongly they disagreed or agreed (responses ranged from 1 to 4) with a list of items. The four items in this analysis included "I see myself as part of the campus community," "Faculty showed concern about my progress," "I feel valued at this institution," and "At least one faculty member has taken an interest in my development."

Outcomes. The predicted state of the students' biomedical major at the end of the first year of college was the outcome of the modeling. Thus, the dependent variable in this analysis

is an indicator of student change regarding the biomedical major. Students reported their major at both time points. Reported majors were grouped into three overall categories of majors based on the list of biomedical majors that are the focus of the Enhance Diversity study, "Biomed-basic science (e.g., biology, chemistry)", engineering with biomedical interest, "Biomed-social science (e.g., psychology, sociology)," and "Non-biomedical (e.g., history)." The major reported at the start of the first year was compared to the major at the end of the first year to assess changes in the major. Students were categorized into the following groups: Persisted in biomedical major, Pushout of a biomedical major, Became a biomedical major, and Never a biomedical major (reference group). In particular, if there was a change in majors during the freshman year, we recorded whether students Became a or were Pushed out of a biomedical major. If there was no change, we recorded whether their major was biomedical or not (hence, the Never and Persisted in as groups).

Because student's major at both time points is critical to defining the outcome, it was also important to determine whether there was bias in the longitudinal sample based on major. For instance, it could be that students pushed out of biomedical majors in the first year were less likely to complete the follow-up survey in the Spring. The distribution of the 3-category biomedical major in the Fall survey for students in the study sample was compared to that for students who only completed the Fall survey. The distributions were not meaningfully different (Cramer's V = 0.09).

Statistical analysis

Data processing and descriptive analysis. The data was checked for valid observations and missing values as a first step. Eight of the 20 variables included in the modeling had missing values, ranging from 0.5% to 44.5%. The missing data did not appear systematic, such as the drop-out of entire sections of the survey skipped by a large group of respondents. Instead, the missing data appears random across individuals and with regard to similar questions. Given the missing value pattern is deemed missing at random, a multiple chain imputation model is applied to correct the missing data gaps [66]. The method used was a categorical multivariate model that takes into account adjacent information about the variables for imputing the data and fits across multiple iterations. The variables reached adequate convergence as evidenced by a visual inspection of the plots showing convergence for each level of the outcome category (pushed out, persisting in, never, and to a biomedical major) and for the mean and standard deviation of each level for all variables across 30 iterations. This plot is available in the appendix for review and demonstrates that the model is consistent across the iteration, and, hence, stable.

Following the data imputation, a summary table of the key hallmarks [30], student attributes, and institutional attributes was produced. All tables have measures grouped by biomedical major outcome, and univariate statistical tests are provided in Table 2, reported as a p-value. Pushout is common, 2140/7252 (30%) of students were pushed out. Table 2 demonstrates significant differences across the biomedical major groups with the major indicators of biomedical education progress. In general, at the end of their first year of college, the pushout cohort had lower levels of science identity, research self-efficacy, and pursuit of science careers that are practically and statistically significant. A sense of belonging in science was not lower for the pushout group.

Table 3 presents the results of the student-based characteristics. These also indicate differences across the major outcome levels.

Statistical modeling. We utilized a multinomial model to assess the associations between the set of predictor variables and all levels of the response variable, biomedical major status, at

Table 2. Summary statistics and univariate tests of key hallmarks by outcome group at the end of the first year.

	Never, N = 3,549	Persisted, N = 1,154	Pushed out, N = 2,140	Became, N = 409	P-value
Science Identity	56 (33, 71)	53 (33, 71)	47 (33, 71)	53 (33, 71)	<0.001
Science Self-Efficacy	49 (14, 72)	47 (14, 72)	43 (14, 72)	47 (19, 72)	<0.001
Sense of Belonging	50 (24, 66)	49 (24, 66)	51 (24, 66)	51 (31, 66)	<0.001
Science Career	4 (3, 5)	4 (2, 4)	2 (1, 3)	4 (3, 4)	<0.001

Kruskal-Wallis rank sum test; Fisher's Exact Test for Count Data with simulated p-value (based on 2000 replicates); Medians and interquartile ranges are reported

https://doi.org/10.1371/journal.pone.0312862.t002

the end of the first year of college. A similar subpopulation analysis was performed for only the pushout versus persisted biomedical major outcomes. Both are discussed in the following section. We chose to present both the multinomial model (with all possible outcomes for biomedical majors including Persisted in, Pushout from, and Became vs. Never) so that a holistic picture of the data is presented as well as the binomial model (with only Persisted in and Pushout from included) for a more accessible depiction of the data. Readers will note that some of the comparisons for the multinomial model are not identical to the binomial model results. This is to be expected since the binomial data omitted 3,958 observations out of the total 7,252 observations used for the multinomial model. The omission of the full data could bias results and having both models provides a stronger basis for evaluating evidence.

The predictors in these models are sociodemographic characteristics (age, financial status, socioeconomic status, native language, race and ethnicity, sex, LGBTQ status, science identity,

Table 3. Student demographics across biomedical major outcomes.

	Never, N = 3,549	Persisted, N = 1,154	Pushed out, N = 2,140	Became, N = 409	P-value
<20 years old	2,063 (96%)	3,451 (97%)	1,120 (97%)	400 (98%)	0.2
Sex					< .001
Male	559 (26%)	1,107 (31%)	367 (32%)	120 (29%)	
Female	1,581 (74%)	2,442 (69%)	787 (68%)	289 (71%)	
LGBTQ					< .001
non-LGBTQ	1,765 (82%)	3,156 (89%)	1,027 (89%)	358 (88%)	
LGBTQ	375 (18%)	393 (11%)	127 (11%)	51 (12%)	
Native English speaker					< .001
No	265 (12%)	544 (15%)	213 (18%)	53 (13%)	
Yes	1,875 (88%)	3,005 (85%)	941 (82%)	356 (87%)	
No concern ability pay for college	384 (18%)	641 (18%)	244 (21%)	42 (10%)	< .001
Pell grant recipient	844 (39%)	1,462 (41%)	490 (42%)	96 (23%)	< .001
Race/ethnicity					
Asian	341 (16%)	877 (25%)	247 (21%)	51 (12%)	< .001
American Indian/Alaskan Native	5 (0.2%)	9 (0.3%)	4 (0.3%)	0 (0%)	.8
Black/African American	256 (12%)	599 (17%)	216 (19%)	37 (9%)	< .001
Hispanic/ Latino	457 (21%)	676 (19%)	249 (22%)	60 (15%)	.004
Multiracial/AIAN	341 (16%)	448 (13%)	168 (15%)	48 (12%)	.004

Prior to modeling the data, we assessed the data for representativeness by comparing the student characteristics, including gender, age, Pell Grant recipients, and race and ethnicity, to the parallel institutional-level characteristics. The goodness-of-fit tests confirm that our sampled data reflects the broader demographics at the included institutions. Finally, association tests demonstrate that institutional factors differ across the major outcomes. All three of these tables demonstrate a univariate association between multiple student-based hallmarks, demographic variables, and institutional factors and merit further modeling to understand how they impact persistence in a biomedical major among first-year students.

https://doi.org/10.1371/journal.pone.0312862.t003

research self-efficacy, sense of belonging), institutional characteristics (HBCU, MSI, Pell %) and undergraduate student experiences (faculty feedback, felt isolated on campus, felt valued in class, faculty encouraged me, felt part of the campus community, faculty were concerned about my progress, found a balance between academic and family responsibilities, felt valued at the institution, valued diversity of opinions, faculty showed interest, and sense of community with students). All single multiple-response option items were entered into the analytic model as numerical variables.

The model-building process used the following step-down strategy: First, a full model was fit, and variance inflation factors were computed for each predictor in the model. Second, factors with three or more nominal or ordinal levels were tested for conditional association with biomedical major outcome using global likelihood ratio tests (LRTs). This approach ensures that the overall association holds prior to testing individual levels, thereby protecting against inflated type I errors. A follow-up analysis on the model-based marginal means transformed into odds ratios investigated these factors further, as described in detail in the following section. All analyses were performed in *R* using the *nnet* [67] and *emmeans* packages [68].

Analysis of institutional environment factor modifiers. To explore whether first-year experiences predict any differences by key sociodemographic predictors, we examined the relationships between independent variables and the primary outcome (biomedical major status at the end of the first year of college) while controlling for important student characteristics such as science identity, research self-efficacy and items related to sense of belonging as well as demographic features. Using the full model, we conducted stratified analyses for significant modifiers. In particular, we investigated specific differences at each level of that modifier while holding all other variables in the model constant at their mean values. When differences in the biomedical major outcome were not indicated as impacted by the modifier as reported in the global test, we did not look for differences within these groups. This approach ensures that type I error rates are not compounded in the overall modeling process. Moreover, a second layer of protection is utilized by employing Tukey adjustments for the pairwise differences of the relevant computed odds ratios at specific levels of the institutional factors, provided there are no straightforward methods for diagnosing model fit in multinomial models [69]. Thus, diagnostics are run on separate logistic regression models for levels of the outcome Pushed out, Became, and Never a biomedical major with reference level Persisted in biomedical major. The model for pushed out versus persisted is reported in the results section due to the importance of these two biomedical major outcome levels and to investigate whether the same association exists if we restrict the analysis to just the cohort of students who persisted or were pushed out of a biomedical major.

Results

Overall, individuals pushed out of biomedical majors were more often male sex, Pell grant recipients, non-native English speakers, Black and Latinx race and ethnicity, and less often Asian. Model results indicate that a number of institutional environment- and student-based characteristics affect biomedical major status at the end of the first year of college. In the full model, including all available covariates, none of the variance inflation factors (VIF) exceeded 5, showing little evidence of multicollinearity. Hence, each variable in the model contributes a unique explanation of the variance in the outcome. Model deviance tests indicate a better fit of the full model when compared to the null model, fitting only non-mutable student characteristics (LRT = 363.1, df = 33, $p<0.0001$). This provides evidence of a superior fit of the full model and confirms that the mutable factors influence biomedical major outcomes, and should be considered further in the model. S1 Table provides overall odds ratio estimates and 95%

confidence intervals for each level of the biomedical major outcome variable compared to the assumed reference level, Never a biomedical major. The table also includes the parallel univariate responses that do not control for other influences in the model.

Multinomial model results

Student and institutional factors associated with biomedical major status at the end of the first year of college are summarized below, with complete results in S1 Table. In all subsections, the model results are presented to compare particular levels of the possible outcomes (i.e., Persisted in, Pushed out, Became a, or Never a biomedical major) for student and institutional factors. The possible outcomes are separated using these levels for ease of presentation, but all results are presented from the full multinomial model that includes all institutional and student-based covariates. This section summarizes the most salient results using Never a biomedical major as a reference group for the full model. These results broadly represent the odds of either Persisting in biomedical or being Pushed out of biomedical major with a neutral level (Never a biomedical major) as a reference, providing a snapshot of first-year biomedical majors and the tendency to Persist in or be Pushed out.

In S2 Table, the binomial logistic regression model results are presented for a subset of the data comparing Persisting in versus Pushed out of a biomedical major. These results provide insight into how first-year in biomedical majors stay in their majors over the first year. The following two sections outline the student and institutional factors, indicating the biomedical outcomes being compared for each factor. The outcomes are noted in parentheses following the odds ratio (OR) abbreviation, indicating the odds of being pushed out of a biomedical major. For this comparison, we use the full multinomial model results alongside results derived from a simple logistic regression model that only includes observations where the students either Persisted in or were Pushed out from a biomedical major. When the results differed between the univariate and multivariate tests provided in S1 and S2 Tables, this is denoted by a hashtag (#). When the results are statistically significant at 5% or 1% this is denoted via a single or double asterisk (* or **), respectively.

Student factors. Using the full multinomial model with all biomedical major outcome levels, student-~~t~~ level factors affecting Persisting in vs Never a biomedical major include science identity (OR = 0.57 (0.51, 0.63), p<0.001); research self-efficacy (OR = 1.17 (1.07, 1.28), p<0.001); intention to pursue a scientific career (OR = 0.86 (0.79, 0.94), p<0.001); being a native English speaker (OR = 0.73 (0.60, 0.88), p = 0.001), or having a professional degree planned (OR = 0.59 (0.48, 0.73), p<0.001).

Factors impacting Pushout vs Never a biomedical major include science identity (OR = 0.51 (0.46, 0.57), p<0.001); intention to pursue a scientific career (OR = 0.40 (0.37, 0.44), p<0.001); being LGBTQ (OR = 1.41 (1.16, 1.72), p<0.001); Asian (OR = 0.46 (0.38, 0.57), p<0.001); Black or African American (OR = 0.63 (0.48, 0.83), p = 0.001); Latinx (OR = 0.65 (0.52, 0.81), p<0.001); having a graduate degree plan (OR = 0.56 (0.47, 0.66), p<0.001), and professional degree planned (OR = 0.21 (0.17, 0.25), p<0.001).

Using the binomial logistic model with only Pushout and Persisting as biomedical major outcomes (see S2 Table) shows that the following factors increase the odds of Pushout vs Persisting in a biomedical major: being LGBTQ (OR = 1.42 (1.11, 1.82), p = 0.005) and a native English speaker (OR = 1.29 (1.02, 1.64), p = 0.036). Student factors decreasing Pushout vs Persisting in a biomedical major include: research self-efficacy (OR = 0.82 (0.74, 0.90), p<0.001), intention to pursue a science career (OR = 0.50 (0.45, 0.55), p<0.001), being Asian (OR = 0.50 (0.39, 0.65), p<0.001), Black or African American/~~Black~~ (OR = 0.57 (0.41, 0.80), p = 0.001), Latinx (OR = 0.62 (0.47, 0.81), p<0.001), multiracial/AIAN (OR = 0.69 (0.53, 0.90), p = 0.006),

and having graduate (0.60 (0.49, 0.73), p<0.001) or professional degree planned (OR = 0.35 (0.27, 0. 44), p<0.001),

Institutional factors. Following the same approach used with student factors above, we present the multinomial model results first and follow with the binomial model results. Factors affecting Persisting in vs Never being a biomedical major include: being at a HBCU (OR = 1.55 (1.20, 2.02), p<0.001), minority-majority institution (OR = 1.28 (1.09, 1.50), p = 0.002), having faculty concerned about progress (OR = 1.15 (1.06, 1.24), p = 0.001), feeling valued at their institution (OR = 0.89 (0.81, 0.97), p = 0.007), having a sense of community with students (OR = 1.12 (1.02, 1.22), p = 0.012), or having conflicts with job responsibilities (OR = 1.09 (1.02, 1.17), p = 0.012).

Factors impacting Pushout versus Never a biomedical major include: being at a HBCU (OR = 1.47, (1.11, 1.95), p = 0.008) or a minority-majority institution (OR = 1.90 (1.63, 2.22), p<0.001), having interactions with graduate students (OR = 0.83 (0.72, 0.95), p = 0.007), feeling isolated on campus (OR = 1.12 (1.04, 1.22), p = 0.003), faculty showing interest (OR = 1.17 (1.08, 1.27), p<0.001), and conflict between school and job (OR = 1.08 (1.01, 1.16), p = 0.034).

In the binomial model only comparing students in the biomedical majors, the following institutional factors impact odds of Pushout vs Persisting in a biomedical major: minority-majority institution (OR = 1.57 (1.30, 1.89), p<0.001), feeling valued at institution (OR = 1.21 (1.09, 1.35), p<0.001), and faculty interest in development (OR = 1.20 (1.09, 1.32), p<0.001). Fig 2 presents the combined forest plots of the binomial logistic model for just the pushout and persisting in a biomedical major outcomes for both univariate and multivariate results.

Discussion

This study examined the experience of over 7,000 first-year, full-time freshmen enrolled at universities with biomedical majors. The outcome of interest was whether a student Persisted in, was Pushed out of, Became a, or Never was a biomedical major, and multiple factors were included in the model to detect associations with this outcome. All outcome categories were included in the analysis to build a model based on the full cohort, however, we focus this discussion on whether the students persisted or were pushed out of biomedical majors. Examining these associations with a large and diverse group of students in their first year of college is particularly important, given that pushout is critical in a nascent undergraduate career. To frame these results, it is important to note that the data are from the very beginning of the NIH BUILD program [70] and are not a reflection of the efficacy of the program but rather, contextually, a description of the dynamics of pushout at these institutions in the pre-NIH BUILD period.

Self-perceptions such as science identity, research self-efficacy, and academic self-concept contribute to students' persistence in biomedical majors [39, 41, 59, 71]. Science identity is predictive of the choice to major in the sciences, [38] the commitment to a science career [31, 35, 36] entrance into a graduate science program, [37] and post-graduation employment as a scientist [39]. Our results support the findings related to science-identity. However, research self-efficacy was not related to pushout or becoming a biomedical major compared to non-biomedical major cohorts. This is a novel finding, most likely due to the nuanced classification of biomedical major outcomes with four distinct levels (pushout from, persist in, became a, and never a biomedical major).

Faculty and staff responsiveness [72] and developing a supportive and caring environment areis related to student persistence in biomedical [73]. Specifically, for first-year students, enrolling in math and science courses [22, 74] and joining an academic club or organization [2] are significant indicators of first-year persistence in biomedical majors. A 2013 National

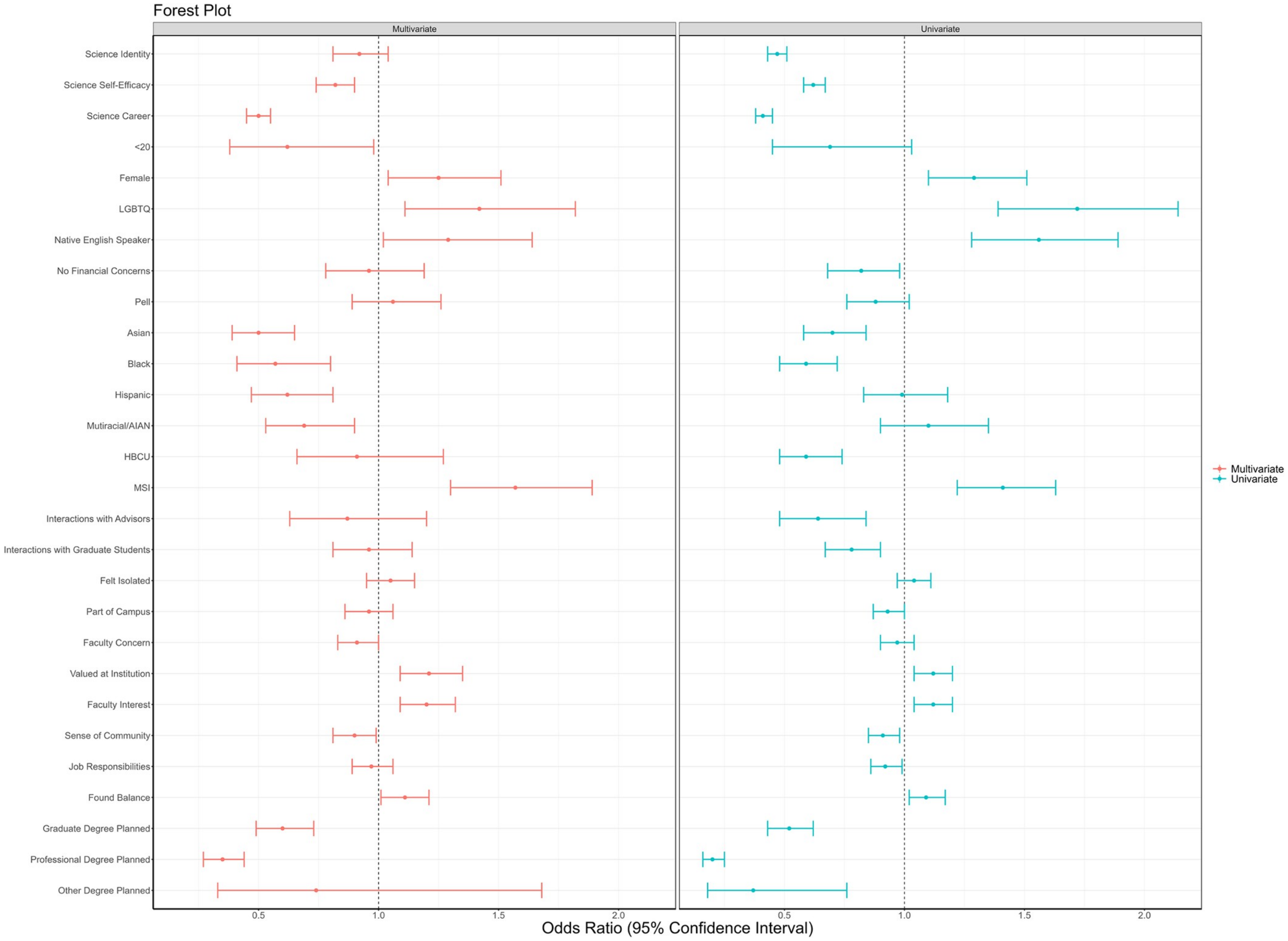

Fig 2. Forest plot of logistic regression model for pushout versus persist.

https://doi.org/10.1371/journal.pone.0312862.g002

Center of Education Statistics (NCES) report found that from 2004–2009, 48% of biomedical majors switched to a non-biomedical major or left a biomedical field by leaving college before earning a degree [17]. This implies that potential biomedical majors become discouraged well before they can join the workforce. Research suggests that undergraduates who are pushed out are often highly qualified college entrants who are disproportionately women and those from minoritized race and ethnicity groups [75]. While the cause for the pushout is multifaceted, some research suggests that feeling unwelcome, [73] isolation and alienation, the nature and quality of science teaching, and the "culture" within the sciences [76, 77] may account for some of the attrition. Our results confirm that feeling isolated increases the odds of pushout of biomedical majors, feeling part of the student community decreases the odds of pushout, and faculty showing concern and feeling valued increases persistence in biomedical majors.

Our results suggest that several additional individual and core institutional factors are associated with pushout from a biomedical major. At the student level, participants who were more likely to experience pushout versus those who were never a biomedical major reported higher levels of feeling isolated, had job conflicts with school, and expressed faculty showing interest in progress. Notably, students who perceived higher levels of faculty interest in their academic progress were less likely to persist [72, 73].

Our study results highlight the role of students' sense of isolation and satisfaction with their community in shaping their ongoing persistence in biomedical majors. These results align and extend the findings of other studies that identified isolation and satisfaction with the student community as important factors to consider for persistence and pushout for overall student populations [29, 33, 48, 49, 78] and biomedical majors. [33, 53, 57] However, our study is the first to confirm these findings in a multi-site cohort of diverse institutions with a large sample size (n = 7,252) suggesting that students' feelings of isolation and sense of community are important for students at various colleges and universities, including HBCUs, MSIs, HSIs, and non-MSIs. Notably, study results found that increased student-reported isolation and decreased satisfaction with the campus community are associated with modestly reduced odds (20–30%) of persistence.

Past studies with URG biomedical students have identified a sense of belonging and found it linked to achievement, persistence, and academic engagement for these students, suggesting that biomedical majors feeling a sense of belonging may be a crucial component of URG students' successful attainment of biomedical degrees [33, 46, 57]. Research has also demonstrated that students from URGs have lower overall levels of belonging than students from well-represented populations within these disciplines [53, 79, 80]. However, a sense of belonging may take on heightened importance for URG students because they often feel unwelcome or like they do not belong in biomedical [81]. Notably, discrimination, microaggressions, and low faculty expectations are widespread occurrences on college campuses for URG biomedical students, and these experiences have well-documented negative relationships with a sense of belonging for URG students [82, 83].

This study is the first to identify a faculty-related variable impacting student pushout. Faculty and student interactions have been identified as a critical ingredient for college student success for all students [61] and URG students specifically [60, 62]. However, negative experiences when interacting with faculty are common for many URG students in biomedical majors. The results from a study by Nora and Cabrera [84] indicated that URG students often have negative experiences interacting with faculty while in the classroom. Additionally, research has found that URG students face higher levels of scrutiny in courses and that faculty members hold lower expectations for their performance, [85] suggesting that faculty have a role in the ongoing negative environments many URG college students encounter. Our findings indicate that early faculty interventions, where faculty explicitly express concern to first-year students interested in biomedical majors, may be crucial for persistence and mitigating pushout.

Even though our study encompassed 11 university sites across the United States and broadly represented majority-minority-serving institutions, including historically Black, Hispanic-serving, and American Indian-serving colleges, several limitations are worth noting. First, the campuses represented in the study may inherently provide social and other support to historically URG students. Thus, the findings may only generalize to universities and colleges that are similar in profile to the ones included in this study. Relatedly, all campuses from which the survey participants were drawn are sites of the NIH-funded BUILD Initiative. Although the survey population includes both BUILD and non-BUILD students, BUILD institutions may have unique characteristics that shape the experiences of first- year students. It is important to note that this survey was delivered very early in the NIH BUILD grant and may reflect the early performance of the NIH BUILD program as a whole. As a result, social isolation, lack of satisfaction with the campus community, and lack of faculty concern experienced on other campuses may have an even more deleterious effect, with a larger effect on pushout.

Second, the study relied on the TFS survey, which had only a 25–30% response rate and thus may not adequately represent all first-year students at the institutions or in the United

States. Nonetheless, as noted in a recent analysis by Norris et al. [64] the findings provide a large sample of first year students across several institution types with wide-ranging diversity. One can view these analyses as a baseline analysis of an ongoing cohort. Still, the cross-sectional design reduces the ability to conclude that the institutional environment and student characteristics lead to the outcomes. Importantly, sense of belonging is measured only at the end of the first year of college.

Third, although our analyses controlled for several confounders, including sex, age, and race and ethnicity, there may have been sources of unmeasured confounding. For example, we did not have measures to include the exact nature, ease of access, utilization, and types of campus support services available to biomedical or other majors, nor did we have access or measures of the specific faculty members and why they expressed concern or the frequency of this contact. Future studies could examine these support services, assess faculty interaction in more detail, and gain more insight into the role of additional campus support in the persistence and pushout first year biomedical students.

Finally, some of the factors isolated as salient come from previously utilized psychometrically validated scales. However, our study was limited as data were not uniformly available across the surveys we considered. Future studies should examine these scales to better understand these phenomena.

A major strength of our study is the large sample size from 11 campuses across the United States with diverse student populations. Further, we emphasize that identifying the effects on the first year of study is one of this paper's strongest and most important points. We identified an early critical period in an undergraduate timeline that can lead to a large pushout of biomedical majors. Developing, testing, and expanding early interventions for this group of students is crucial. Several biomedical-focused student-centered programs, including NIH-BUILD programs, Meyerhoff Scholars, and UC Berkley Biology Scholars Programs, provide interventions and support to recruit and retain students to biomedical majors at different stages of their undergraduate careers. Part of our analyses indicate that demonstration of faculty concern is impactful. Developing and implementing interventions incorporating timely and regular expression of faculty concern to first-year biomedical students may prevent pushout.

Our study diverges from past studies on this topic by suggesting that increasing persistence or switching to a biomedical major in the first year is not the converse of pushout. In other words, increasing versus decreasing one influential factor may not necessarily lead to persistence versus pushout. Rather, ~~there are likely~~ distinct factors likely contribute to persistence, pushout, or becoming a biomedical major, and each should be studied independently. Results suggest several actionable interventions that can be initiated to increase recruitment to and persistence in biomedical majors and mitigate pushout. Institutional-level, early -interventions can be adopted by university administrators and stakeholders, including provosts, deans, department chairs, DEI committees, faculty, and staff, to increase student persistence in biomedical majors and reduce pushout.

This study is among the first to identify the first year as a critical window to prevent pushout from biomedical majors for historically URG students. The identified student-based factors, isolation, and satisfaction with the campus community, are modifiable and can be addressed through intentional efforts at the campus level. Additionally, faculty demonstration of concern for students is a faculty-student interaction that can be addressed at an institutional level. Study results provide a rationale to prioritize institutional transformation through early college career biomedical student interventions and suggest that doing so may increase biomedical major persistence in 20–30% of students.

Implications

While we note that these findings align with previous research, a notable issue is the magnitude at which these data show the pushout of URG students, indicating the large extent of this problem nationwide. The identification of both individual student and institutional factors as potential influences on pushout underscores the need for institutions to address factors within their power to effectively mitigate the pushout identified in this paper so that the environment can allow for change at the individual level. The unexpected findings that students were pushed out instead of persisting in biomedical majors more often at majority-minority institutions, when a student reported feeling valued and when faculty are concerned for progress highlights the need for an experimentally designed detailed evaluation of interventions at HBCU and minority-majority institutions to better understand the intricacies of these influences. This could inform larger- scale interventions to other institutions, nationwide.

Supporting information

S1 Table. Multinomial regression model results.
(DOCX)

S2 Table. Logistic regression model results.
(DOCX)

Acknowledgments

We want to thank members of the DPC for their steadfast contributions and all Enhance Diversity Study participants for their willingness to contribute. We greatly appreciate the support of the CEC, which conducted the data acquisition process. The authors also wish to acknowledge the contributions to this work from colleagues who provided early reviews and advice, including Ellen Lopez (University of Alaska Fairbanks), Clair Wilkins-Green (Xavier University of Louisiana), and Cleo Hughes-Darden (Morgan State University). We want to acknowledge all members of the NIH BUILD consortium team—instructors, staff, and mentors—for their collaboration at 10 sites across the country.

References

1. APPG on Diversity and Inclusion in STEM. Inquiry into Equity in STEM Workforce: Final Report. London: 2021 July. Report No.
2. Chang MJ, Cerna O, Han J, Saenz V. The contradictory roles of institutional status in retaining underrepresented minorities in biomedical and behavioral science majors. Review of Higher Education. 2008; 31(4):433–64.
3. National Academy of Sciences, National Academy of Engineering, Institute of Medicine. Expanding underrepresented minority participation: America's science and technology talent at the crossroads. Washington, DC: National Academies Press 2011.
4. Hong L, Page SE. Groups of diverse problem solvers can outperform groups of high-ability problem solvers. Proceedings of the National Academy of Sciences. 2004; 101(46):16385–9. https://doi.org/10.1073/pnas.0403723101 PMID: 15534225
5. Valantine HA, Collins FS. National Institutes of Health addresses the science of diversity. Proceedings of the National Academy of Sciences. 2015; 112(40):12240–2. https://doi.org/10.1073/pnas.1515612112 PMID: 26392553
6. Woolston C. Minority representation in US science workforce sees few gains. Nature. 2021; 592 (7856):805–6. https://doi.org/10.1038/d41586-021-01089-6.
7. Center for Institutional Data Exchange and Analysis. 1999–2000 SMET retention report. Norman, OK: University of Oklahoma; 2000.

8. Eagan MK Jr, Hurtado S, Chang MJ, Garcia GA, Herrera FA, Garibay JC. Making a difference in science education: The impact of undergraduate research programs. American educational research journal. 2013; 50(4):683–713. https://doi.org/10.3102/0002831213482038 PMID: 25190821

9. Higher Education Research Institute. Degrees of success: Bachelor's degree completion rates among initial STEM majors. Los Angeles: Higher Education Research Institute; 2010.

10. Boyle P. U.S. Physician Shortage Growing 2020. Available from: www.aamc.org/news-insights/us-physician-shortage-growing.

11. Haddad L, Annamaraju P, Toney-Butler T. Nursing Shortage. StatPearls (Internet). Treasure Island, FL: StatPearls Publishing; 2023.

12. Garrison H. Underrepresentation by race–ethnicity across stages of US science and engineering education. CBE—Life Sciences Education. 2013; 12(3):357–63.

13. Sheltzer JM, Smith JC. Elite male faculty in the life sciences employ fewer women. Proceedings of the National Academy of Sciences. 2014; 111(28):10107–12. https://doi.org/10.1073/pnas.1403334111 PMID: 24982167

14. Chang MJ, Sharkness J, Hurtado S, Newman CB. What matters in college for retaining aspiring scientists and engineers from underrepresented racial groups. Journal of Research in Science Teaching. 2014; 51(5):555–80.

15. National Science Foundation. Women, Minorities, and Persons with Disabilities in Science and Engineering: 2017, Table 2–8. Arlington, VA: National Science Foundation; 2017.

16. Riegle-Crumb C, King B, Irizarry Y. Does STEM stand out? Examining racial/ethnic gaps in persistence across postsecondary fields. Educational Researcher. 2019; 48(3):133–44. https://doi.org/10.3102/0013189x19831006 PMID: 39005239

17. Chen X. STEM Attrition: college students' paths into and out of STEM fields. Statistical Analysis Report. NCES 2014–001. National Center for Education Statistics. 2013.

18. Braun DC, Clark MD, Marchut AE, Solomon CM, Majocha M, Davenport Z, et al. Welcoming deaf students into STEM: Recommendations for university science education. CBE—Life Sciences Education. 2018; 17(3):es10. https://doi.org/10.1187/cbe.17-05-0081 PMID: 30142044

19. Estrada M, Burnett M, Campbell AG, Campbell PB, Denetclaw WF, Gutiérrez CG, et al. Improving underrepresented minority student persistence in STEM. CBE—Life Sciences Education. 2016; 15(3): es5. https://doi.org/10.1187/cbe.16-01-0038 PMID: 27543633

20. Maccalla N, Gutierrez A, Zhong S, Wallace S, McCreath H. Evaluation of Post-secondary Student Outcomes: Underrepresented (URG) and Well-Represented (WRG) Group Variable Construction in the Enhance Diversity Study using the November 2019 NIH Guidelines. UCLA Coordinating and Evaluation Center, 2020.

21. Griffith AL. Persistence of women and minorities in STEM field majors: Is it the school that matters? Economics of Education Review. 2010; 29(6):911–22.

22. Weston TJ. Patterns of switching and relocation. In: Seymour E, Hunter A-B, editors. Talking about leaving revisited. Switzerland: Springer Nature; 2019. p. 55–85.

23. Wilton M, Gonzalez-Niño E, McPartlan P, Terner Z, Christoffersen RE, Rothman JH. Improving academic performance, belonging, and retention through increasing structure of an introductory biology course. CBE—Life Sciences Education. 2019; 18(4):ar53. https://doi.org/10.1187/cbe.18-08-0155 PMID: 31675276

24. Gibbs KD Jr, McGready J, Bennett JC, Griffin K. Biomedical science Ph. D. career interest patterns by race/ethnicity and gender. PloS one. 2014; 9(12):e114736. https://doi.org/10.1371/journal.pone.0114736 PMID: 25493425

25. Rosenzweig EQ, Hecht CA, Priniski SJ, Canning EA, Asher MW, Tibbetts Y, et al. Inside the STEM pipeline: Changes in students' biomedical career plans across the college years. Science Advances. 2021; 7(18):eabe0985. https://doi.org/10.1126/sciadv.abe0985 PMID: 33931444

26. Beasley MA. Opting Out: Losing the Potential of America's Young Black Elite: University of Chicago Press; 2012.

27. Beasley MA, Fischer MJ. Why they leave: The impact of stereotype threat on the attrition of women and minorities from science, math and engineering majors. Social Psychology of Education. 2012; 15 (4):427–48.

28. Steele CM, Aronson J. Stereotype threat and the intellectual test performance of African Americans. Journal of personality and social psychology. 1995; 69(5):797. https://doi.org/10.1037//0022-3514.69.5.797 PMID: 7473032

29. O'Keeffe P. A sense of belonging: Improving student retention. College Student Journal. 2013; 47 (4):605–13.

30. McCreath HE, Norris KC, Calderón NE, Purnell DL, Maccalla NM, Seeman TE, editors. Evaluating efforts to diversify the biomedical workforce: the role and function of the Coordination and Evaluation Center of the Diversity Program Consortium. BMC proceedings; 2017: Springer.

31. Camacho TC, Vasquez-Salgado Y, Chavira G, Boyns D, Appelrouth S, Saetermoe C, et al. Science Identity among Latinx Students in the Biomedical Sciences: The Role of a Critical Race Theory-Informed Undergraduate Research Experience. CBE life sciences education. 2021; 20(2):ar23. Epub 2021/05/04. https://doi.org/10.1187/cbe.19-06-0124 PMID: 33938764; PubMed Central PMCID: PMC8734380.

32. Ballen CJ, Wieman C, Salehi S, Searle JB, Zamudio KR. Enhancing diversity in undergraduate science: Self-efficacy drives performance gains with active learning. CBE life sciences education. 2017; 16(4): ar56, 1–6. Epub 2017/10/22. https://doi.org/10.1187/cbe.16-12-0344 PMID: 29054921; PubMed Central PMCID: PMC5749958.

33. Strayhorn TL, Lo M-T, Travers CS, Tillman-Kelly DL. Assessing the relationship between well-being, sense of belonging, and confidence in the transition to college for Black male collegians. Spectrum: A Journal on Black Men. 2015; 4(1):127–38.

34. Carlone HB, Johnson A. Understanding the science experiences of successful women of color: Science identity as an analytic lens. Journal of Research in Science Teaching: The Official Journal of the National Association for Research in Science Teaching. 2007; 44(8):1187–218.

35. Chemers MM, Zurbriggen EL, Syed M, Goza BK, Bearman S. The role of efficacy and identity in science career commitment among underrepresented minority students. Journal of Social Issues. 2011; 67 (3):469–91.

36. Estrada M, Woodcock A, Hernandez PR, Schultz P. Toward a model of social influence that explains minority student integration into the scientific community. Journal of Educational Psychology. 2011; 103 (1):206–22. https://doi.org/10.1037/a0020743 PMID: 21552374

37. Merolla DM, Serpe RT. STEM enrichment programs and graduate school matriculation: the role of science identity salience. Social psychology of education: an international journal. 2013; 16(4):575–97. Epub 2014/03/01. https://doi.org/10.1007/s11218-013-9233-7 PMID: 24578606; PubMed Central PMCID: PMC3932434.

38. Robinson KA, Perez T, Carmel JH, Linnenbrink-Garcia L. Science identity development trajectories in a gateway college chemistry course: Predictors and relations to achievement and STEM pursuit. Contemp Educ Psychol. 2019; 56:180–92. Epub 2019/03/12. https://doi.org/10.1016/j.cedpsych.2019.01.004 PMID: 30853745; PubMed Central PMCID: PMC6404963.

39. Robinson KA, Perez T, Nuttall AK, Roseth CJ, Linnenbrink-Garcia L. From science student to scientist: Predictors and outcomes of heterogeneous science identity trajectories in college. Dev Psychol. 2018; 54(10):1977–92. Epub 2018/09/21. https://doi.org/10.1037/dev0000567 PMID: 30234346; PubMed Central PMCID: PMC6152842.

40. Stets JE, Brenner PS, Burke PJ, Serpe RT. The science identity and entering a science occupation. Social Science Research. 2017; 64:1–14. https://doi.org/10.1016/j.ssresearch.2016.10.016 PMID: 28364837

41. Bong M. Academic motivation in self-efficacy, task value, achievement goal orientations, and attributional beliefs. Journal of Educational Research. 2004; 97(6):287–98.

42. Brown SD, Lent RW, Larkin KC. Self-efficacy as a moderator of scholastic aptitude-academic performance relationships. Journal of Vocational Behavior. 1989; 35(1):64–75.

43. Chemers MM, Hu L-t, Garcia BF. Academic self-efficacy and first year college student performance and adjustment. Journal of Educational Psychology. 2001; 93(1):55–64.

44. Connell JP, Wellborn JG. Competence, autonomy, and relatedness: A motivational analysis of self-system processes. In: Gunnar MR, Sroufe LA, editors. Self Processes and Development: Lawrence Erlbaum Associates, Inc.; 1991.

45. Furrer C, Skinner E. Sense of relatedness as a factor in children's academic engagement and performance. Journal of Educational Psychology. 2003; 95(1):148–62.

46. Garcia GA, Hurtado S. Predicting Latina/o STEM persistence at HSIs and non-HSIs. Paper presented at the American Educational Research Association annual meeting; New Orleans, LA 2011.

47. Hackett G, Betz NE, Casas JM, Rocha-Singh IA. Gender, ethnicity, and social cognitive factors predicting the academic achievement of students in engineering. Journal of Counseling Psychology. 1992; 39 (4):527–38.

48. Hausmann LR, Schofield JW, Woods RL. Sense of belonging as a predictor of intentions to persist among African American and White first-year college students. Research in higher education. 2007; 48:803–39.

49. Johnson DR, Soldner M, Leonard JB, Alvarez P, Inkelas KK, Rowan-Kenyon HT, et al. Examining sense of belonging among first-year undergraduates from different racial/ethnic groups. Journal of College Student Development. 2007; 48(5):525–42.
50. Lent RW, Brown SD, Hackett G. Toward a unifying social cognitive theory of career and academic interest, choice, and performance. Journal of Vocational Behavior. 1994; 45(1):79–122.
51. Lent RW, Brown SD, Larkin KC. Self-efficacy in the prediction of academic performance and perceived career options. Journal of Counseling Psychology. 1986; 33(3):265–9.
52. Osterman KF. Students' need for belonging in the school community. Review of educational research. 2000; 70(3):323–67.
53. Rainey K, Dancy M, Mickelson R, Stearns E, Moller S. Race and gender differences in how sense of belonging influences decisions to major in STEM. Int J STEM Educ. 2018; 5(1):10. Epub 2019/01/12. https://doi.org/10.1186/s40594-018-0115-6 PMID: 30631700; PubMed Central PMCID: PMC6310405.
54. Thomas L. Building student engagement and belonging in Higher Education at a time of change. London, UK: 2012.
55. Tinto V. Constructing Educational Communities: Increasing Retention in Challenging Circumstances. Community College Journal. 1994; 64(4):26–9.
56. Wigfield A, Eccles JS, Schiefele U, Roeser RW, Davis-Kean P. Development of Achievement Motivation. In: Eisenberg N, Damon W, Lerner RM, editors. Handbook of Child Psychology: Social, Emotional, and Personality Development. New Jersey: John Wiley & Sons, Inc.; 2006.
57. Wilson D, Jones D, Bocell F, Crawford J, Kim MJ, Veilleux N, et al. Belonging and academic engagement among undergraduate STEM students: A multi-institutional study. Research in higher education. 2015; 56(7):750–76.
58. Zhang Z, RiCharde RS, editors. Prediction and analysis of freshman retention. AIR 1998 Annual Forum Paper, Minneapolis, MN; 1998.
59. Zimmerman BJ. Self-efficacy: An essential motive to learn. Contemporary educational psychology. 2000; 25(1):82–91. https://doi.org/10.1006/ceps.1999.1016 PMID: 10620383
60. Cole D, Espinoza A. Examining the academic success of Latino students in science technology engineering and mathematics (STEM) majors. Journal of College Student Development. 2008; 49(4):285–300.
61. Lundberg CA, Schreiner LA. Quality and frequency of faculty-student interaction as predictors of learning: An analysis by student race/ethnicity. Journal of College Student Development. 2004; 45(5):549–65.
62. Pascarella ET, Terenzini PT. How College affects Students: Findings and Insights from Twenty Years of Research. San Francisco, CA: Jossey-Bass; 1991.
63. Delaney AM. Why faculty-student interaction matters in the first year experience. Tertiary Education and Management. 2008; 14:227–41.
64. Norris KC, McCreath HE, Hueffer K, Aley SB, Chavira G, Christie CA, et al. Baseline Characteristics of the 2015–2019 First Year Student Cohorts of the NIH Building Infrastructure Leading to Diversity (BUILD) Program. Ethnicity & disease. 2020; 30(4):681–92. Epub 2020/09/30. https://doi.org/10.18865/ed.30.4.681 PMID: 32989368; PubMed Central PMCID: PMC7518523.
65. Hurtado S, Eagan MK, Cabrera NL, Lin MH, Park J, Lopez M. Training future scientists: Predicting first-year minority student participation in health science research. Research in higher education. 2008; 49(2):126–52. Epub 2008/03/01. https://doi.org/10.1007/s11162-007-9068-1 PMID: 23503996; PubMed Central PMCID: PMC3596162.
66. Van Buuren S. Flexible Imputation of Missing Data. Boca Raton, FL: Chapman & Hall/CRC Press; 2018.
67. Venables WN, Ripley BD. Modern Applied Statistics with S-PLUS. 4th ed. New York: Springer Science & Business Media; 2013.
68. Lenth R, Singmann H, Love J, Buerkner P, Herve M. Emmeans: Estimated marginal means, aka least-squares means. R package version 153 2018.
69. Agresti A. Categorical data analysis: John Wiley & Sons; 2012.
70. Hurtado S, White-Lewis D, Norris K. Advancing inclusive science and systemic change: The convergence of national aims and institutional goals in implementing and assessing biomedical science training. BMC proceedings. 2017; 11(Suppl 12):17. Epub 2017/12/04. https://doi.org/10.1186/s12919-017-0086-5 PMID: 31851727; PubMed Central PMCID: PMC5773897
71. Cromley JG, Perez T, Kaplan A. Undergraduate STEM achievement and retention: Cognitive, motivational, and institutional factors and solutions. Policy Insights from the Behavioral and Brain Sciences. 2016; 3(1):4–11.

72. Kuh GD, Kinzie JL, Buckley JA, Bridges BK, Hayek JC. What matters to student success: A review of the literature. National Postsecondary Education Cooperative, Washington, DC, 2006.

73. Christe BL. The importance of faculty-student connections in STEM disciplines. Journal of STEM Education: Innovations and Research. 2013; 14(3):22–6.

74. Paschal J, Taggart A. An examination of the role of first-year college-level mathematics in STEM field major persistence at a Hispanic-serving institution. Journal of Hispanic Higher Education. 2021; 20 (3):297–312.

75. National Academy of Sciences, National Academy of Engineering, Medicine Io. Rising Above the Gathering Storm: Energizing and Employing America for a Brighter Economic Future. Washington, DC: The National Academies Press; 2007.

76. McGee R, Keller JL. Identifying future scientists: predicting persistence into research training. CBE—Life Sciences Education. 2007; 6(4):316–31. https://doi.org/10.1187/cbe.07-04-0020 PMID: 18056303

77. Weston TJ, Seymour E, Koch AK, Drake BM. Weed-out classes and their consequences. In: Seymour E, Hunter A-B, editors. Talking about leaving revisited. Switzerland: Springer Nature; 2019. p. 197–244.

78. Bettencourt GM, Manly CA, Kimball E, Wells RS. STEM degree completion and first-generation college students: A cumulative disadvantage approach to the outcomes gap. The Review of Higher Education. 2020; 43(3):753–79.

79. Johnson DR. Campus racial climate perceptions and overall sense of belonging among racially diverse women in STEM majors. Journal of College Student Development. 2012; 53(2):336–46.

80. Walton GM, Cohen GL. A question of belonging: race, social fit, and achievement. Journal of personality and social psychology. 2007; 92(1):82–96. https://doi.org/10.1037/0022-3514.92.1.82 PMID: 17201544

81. Freeman TM, Anderman LH, Jensen JM. Sense of belonging in college freshmen at the classroom and campus levels. The Journal of Experimental Education. 2007; 75(3):203–20.

82. Chang MJ, Eagan MK, Lin MH, Hurtado S. Considering the impact of racial stigmas and science identity: Persistence among biomedical and behavioral science aspirants. Journal of Higher Education. 2011; 82(5):564–96. https://doi.org/10.1353/jhe.2011.0030 PMID: 23226874

83. Hurtado S, Ruiz Alvarado A. Discrimination and bias, underrepresentation, and sense of belonging on campus. Los Angeles, CA: Higher Education Research Institute; 2015.

84. Nora A, Cabrera AF. The role of perceptions of prejudice and discrimination on the adjustment of minority students to college. Journal of Higher Education. 1996; 67(2):119–48.

85. McGee EO, Martin DB. "You would not believe what I have to go through to prove my intellectual value!" Stereotype management among academically successful Black mathematics and engineering students. American Educational Research Journal. 2011; 48(6):1347–89.
